# Dynamic CT myocardial perfusion without image registration

**DOI:** 10.1038/s41598-022-16573-w

**Published:** 2022-07-23

**Authors:** Logan Hubbard, Shant Malkasian, Sabee Molloi

**Affiliations:** Department of Radiological Sciences, Medical Sciences I, B-140, University of California, Irvine, CA 92697 USA

**Keywords:** Computed tomography, Cardiovascular diseases

## Abstract

The aim of this study was to validate a motion-immune (MI) solution to dynamic CT myocardial perfusion measurement, in the presence of motion without image registration. The MI perfusion technique was retrospectively validated in six swine (37.3 ± 7.5 kg) with a motion-susceptible (MS) perfusion technique performed for comparison. In each swine, varying severities of stenoses were generated in the left anterior descending (LAD) coronary artery using a balloon under intracoronary adenosine stress, followed by contrast-enhanced imaging with 20 consecutive volume scans per stenosis. Two volume scans were then systematically selected from each acquisition for both MI and MS perfusion measurement, where the resulting LAD and left circumflex (LCx) measurements were compared to reference microsphere perfusion measurements using regression and diagnostic performance analysis. The MI (P_MI_) and microsphere (P_MICRO_) perfusion measurements were related through regression by P_MI_ = 0.98 P_MICRO_ + 0.03 (r = 0.97), while the MS (P_MS_) and microsphere (P_MICRO_) perfusion measurements were related by P_MS_ = 0.62 P_MICRO_ + 0.15 (r = 0.89). The accuracy of the MI and MS techniques in detecting functionally significant stenosis was 93% and 84%, respectively. The motion-immune (MI) perfusion technique provides accurate myocardial perfusion measurement in the presence of motion without image registration.

## Introduction

Coronary artery disease (CAD) is the leading cause of mortality in the United States. Fortunately, coronary computed tomography angiography (CTA) can accurately detect or rule out high-grade CAD and is non-inferior to functional testing in patients with low- to intermediate-risk of CAD^1^. That said, CTA cannot assess myocardial perfusion in such low- to intermediate-risk patients^2^, where ischemia-guided coronary revascularization is superior to angiography-guided revascularization^3^. Hence, functional testing such as SPECT and CMR are recommended, but such modalities remain limited as they only provide metrics of relative perfusion^4,5^. Of course, dynamic PET has the highest accuracy for diagnosis of myocardial ischemia^6^, yet, radiotracer availability and cost largely limit its routine application. Thus, the ability of dynamic CT to assess perfusion and ischemia in quantitative terms (mL/min/g) is of clinical merit^7–9^. More importantly, evidence supports the combined or tiered use of coronary CTA with dynamic CT perfusion for morphological *and* physiological assessment of CAD^7–12^. That said, routine use of dynamic CT perfusion remains limited by the added contrast and radiation dose required to measure perfusion. Specifically, dynamic CT techniques rely on ECG-gated scanning of the myocardium over 10 to 20 heart beats. While ECG-gating ensures the same cardiac phase is captured for each scan; unfortunately, beat-to-beat variation and imperfect breath-holding necessitates image registration between consecutive acquisitions. Nevertheless, registration itself inherently alters the myocardial CT number, incurring errors in quantitative perfusion measurement. Moreover, dynamic CT perfusion techniques underestimate perfusion as compared to quantitative PET, especially under hyperemic conditions^13^, where such inaccuracies are further exacerbated by cardiac and respiratory motion despite registration^14^. Hence, a better solution to dynamic CT perfusion measurement remains necessary.

Prior work in first-pass analysis (FPA) dynamic CT perfusion measurement has addressed some of these limitations via a new technique capable of accurate perfusion measurement with only two volume scans, where one volume scan may also double as a coronary CTA^15–18^. Yet, a remaining limitation of this motion-susceptible (MS) FPA technique is its reliance on image registration to minimize motion between the two volume scans of interest.

Hence, this study aimed to validate a motion-immune (MI) solution to FPA dynamic CT perfusion measurement using quantitative microsphere perfusion measurement as the reference standard. The central hypothesis was that accurate myocardial perfusion measurement is feasible with the MI perfusion technique in the presence of motion without image registration.

## Methods

### General methods

The study was performed on six male Yorkshire swine (37.3 ± 7.5 kg). It was approved by the Institutional Animal Care and Use Committee at UC Irvine (IACUC Protocol Number: AUP-22-015) and was carried out in accordance with all relevant regulations, as well as in compliance with the ARRIVE guidelines. In each animal, several different intermediate severity balloon stenoses were generated in the left anterior descending (LAD) coronary artery, after which dynamic imaging was performed. The data were then processed, in the absence of image registration, using the motion-immune (MI) perfusion technique as well as the previously validated motion-susceptible (MS) perfusion technique^15,16^, after which both techniques were compared to reference standard microsphere perfusion measurement. Of note, the raw data used were previously acquired to validate the MS perfusion technique with registration^15^, but the analysis and validation presented in this study are new and independent.

### Motion-immune dynamic CT perfusion theory

Modelling the whole myocardium as one compartment, the average perfusion (P_AVG_) is approximately proportional to the first-pass rate of contrast mass entry into the compartment over time (dM_C_/dt: in grams of Iodine per minute), normalized by the average incoming aortic blood pool contrast concentration (C_IN_: in grams of Iodine per milliliter of blood) and total left ventricular tissue mass (M_T_: in grams), assuming no contrast mass outflow at the time of measurement^15,16^. Importantly, since the mass of Iodine is related to the enhancement of tissue and blood by the same physical constant which cancels in ratio, only the tissue and blood enhancement (in Hounsfield Units, HU) of two whole-heart volume scans acquired at the base and peak of the aortic enhancement (V1 and V2) are needed for perfusion measurement, where V1 is effectively a non-contrast volume scan while V2 is effectively a coronary CTA, as previously validated^15,16^. Differently, however, the integrated enhancement of V2 is determined by summating all myocardial enhancement values within V2. The integrated enhancement of V1 is then approximated by multiplying the average myocardial enhancement of V1 by the total number of myocardial voxels, *n*, within V2. Taken in difference, dM_C_/dt is calculated and normalized by the blood pool contrast concentration (C_IN_: the average aortic enhancement between V1 and V2) and total tissue mass (M_T_: the product of *n*, the voxel size, and the tissue density) to yield the average perfusion (P_AVG_), shown in Eq. (1.1). The average change in myocardial enhancement (ΔHU_AVG_) is then calculated as the average difference in voxel values between the V2 and V1 volume scans. Finally, the average enhancement of V1 is subtracted per-voxel from V2 to estimate the voxel-by-voxel differences in myocardial enhancement between V1 and V2 (ΔHU*). In combination, MI perfusion (P_MI_) in mL/min/g is derived, as described by Eq. (1.2). The assumptions are: (1) that the V2 and V1 myocardium are equivalent in volume, and (2) that the myocardial tissue density of V1 is homogenous since negligible contrast mass has entered the myocardium at the time of V1 acquisition.

1.1 $${P}_{AVG}={M}_{T}^{-1}{C}_{IN}^{-1}\frac{d{M}_{c}}{dt}$$

1.2 $${P}_{MI}={P}_{AVG}\cdot \frac{\Delta H{U}^{*}}{\Delta {HU}_{AVG}}$$

### Animal model

**Figure 1:**
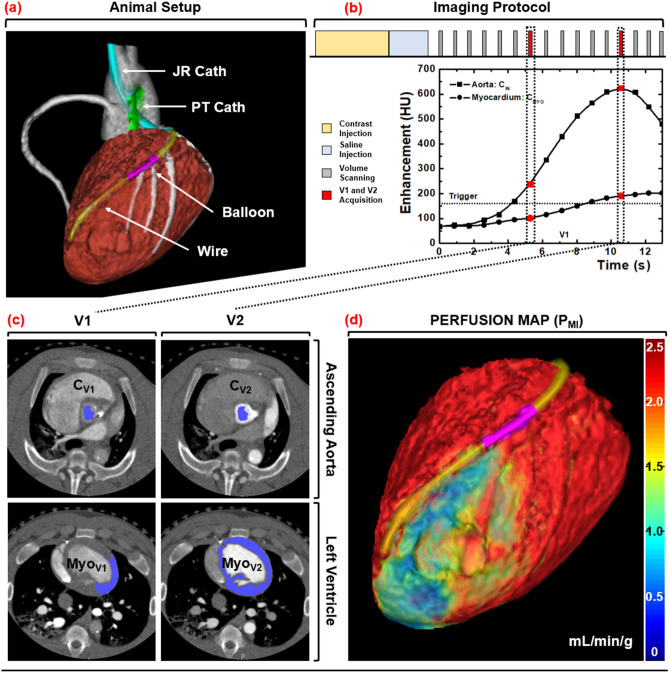
Animal setup, imaging protocol, and image processing scheme. (**a**) Interventional setup displaying the Judkins Right (JR) and Pigtail (PT) catheters, coronary balloon, and pressure wire. (**b**) Dynamic CT imaging protocol, with V1 and V2 denoted in red. (**c**) Semi-automatic segmentation of the aortic blood pool and myocardium. (**d**) MI perfusion map derivation with a distal LAD defect displayed.

Induction of anesthesia was accomplished with Telazol (4.4 mg/kg), Ketamine (2.2 mg/kg), and Xylazine (2.2 mg/kg), followed by maintenance with 1.5–2.5% Isoflurane (Highland Medical Equipment and Baxter). Introducer sheaths (5–7 Fr, AVANTI^®^, Cordis Corporation) were then placed in both femoral arteries, one carotid artery, both femoral veins, and one jugular vein. Using the femoral arterial sheaths, a pigtail catheter was placed into the left ventricular blood pool for injection of microspheres, while a multipurpose catheter was placed into the abdominal aorta for withdrawal of reference blood. Using the carotid arterial sheath, a Judkins right catheter was placed, after which a fractional flow reserve wire (FFR) (PrimeWire, Volcano) was advanced down the LAD. A balloon was then passed into the LAD to generate stenoses with incrementally increasing FFR severities of 1.0, 0.9, 0.8, 0.7, and 0.6 at maximal intracoronary hyperemia (240 µg adenosine/min, Model 55-2222, Harvard Apparatus). Note that intracoronary hyperemia was used to minimize the hypotension and reflex tachycardia characteristic to intravenous adenosine, but limited stress perfusion measurement to the LAD alone. Next, the jugular vein sheath was set up for contrast injection, while the femoral vein sheaths were used for intravenous fluids and medications. Finally, the heart rate, end-tidal CO_2_, pulse oximetry, and arterial-line blood pressure were monitored continuously (SurgiVet, Smiths Medical) and logged every 15 min. Using these metrics, the intravenous fluid drip rate, anesthesia depth, and ventilation settings were adjusted accordingly to maintain the mean-arterial pressure greater than 65 mmHg and pulse oximetry greater than 92% for adequate myocardial perfusion and tissue oxygenation, respectively. The animal setup is shown in Fig. 1a.

### Imaging protocol

For each stenosis, hyperemia was induced and maintained throughout acquisition. For each acquisition, contrast (1 mL/kg, Isovue 370, Bracco Diagnostics) was injected (7 mL/s, Empower CTA, Acist Medical Systems) then chased by saline (0.5 mL/kg at 7 mL/s) with one microsphere color also injected. Dynamic whole-heart volume scanning was then performed at 100 kVp and 200 mA, where each volume scan was acquired as a full projection with a rotation time of 0.35 s and 320 × 0.5 mm collimation (Aquilion One, Canon Medical Systems). After each twenty-scan acquisition, a 15-min delay was observed. Then the stenosis severity was incrementally increased (as described above in 2.3), and the acquisition process was repeated, until all five of the available microsphere colors were used. The 32 cm diameter volumetric CT dose index (${CTDI}_{vol}^{32}$) and size-specific dose estimate (SSDE) were also recorded^19^. The acquisition protocol is shown in Fig. 1b.

### Microsphere protocol

The 15.5 µm diameter NuFlow HydroCoat fluorescent microspheres (IMT Laboratories) were used as the reference standard for quantitative perfusion measurement in mL/min/g^15^. For each image acquisition, 2 mL of unique microsphere color was manually injected into the pigtail catheter then rapidly flushed into the left ventricular blood pool with 5 mL of saline, after which reference blood samples were withdrawn at 10 mL/min over two minutes using a syringe pump (GenieTouch; Kent Scientific). After all the microsphere colors had been injected, each animal was euthanized. Their hearts were surgically removed, and 10-g tissue plugs were extracted from the proximal and distal LAD, as well as from the left circumflex (LCx) perfusion territories. All tissue and blood samples were analyzed by IMT Laboratories.

### Image processing

All volume scans were reconstructed with AIDR 3D (Canon Medical Systems) at 75% of the R-R interval with a voxel size of 0.43 × 0.43 × 0.50 mm. The V1 and V2 volume scans were then systematically selected for analysis^15–18^. For V1, volumetric region growing was used to measure the average enhancement of the aortic blood pool (Vitrea fX version 6.0, Vital Images). For V2, region growing was again used to measure the average enhancement of the aortic blood pool, while segmentation was used to extract and export the entire left ventricular myocardium. All data were then imported into custom in-house software for MI and MS perfusion calculation.

#### MI perfusion calculation

The integrated enhancement of V2 was first determined by summating all the myocardial enhancement values within the segmented left ventricular myocardium. Next, volumetric region growing was used to measure the average enhancement of the lateral wall of the V1 myocardium, which approximated the average enhancement of the total V1 myocardium. The integrated enhancement of V1 was then estimated by multiplying the average enhancement of the lateral wall of the V1 myocardium by the total voxel volume of the segmented myocardium from V2. The total difference in integrated enhancement between V2 and V1 (dM_C_/dt), was then normalized by the average incoming aortic blood pool contrast concentration (C_IN_) and left ventricular myocardium tissue mass (M_T_) to yield the average MI perfusion (P_AVG_MI_). Next, using the V2 segmentation, the average enhancement of the V1 myocardium was subtracted from each voxel of V2 (ΔHU*). Each voxel value was then normalized by the difference in average enhancement between the V2 and V1 myocardium (ΔHU_AVG_) to yield a perfusion ratio map. The average MI perfusion (P_AVG_MI_) was then multiplied by the perfusion ratio map (ΔHU*/ ΔHU_AVG_) to yield voxel-by-voxel MI perfusion measurements (P_MI_).

#### MS perfusion calculation

The segmented myocardium of V2 was applied as a binary mask to segment the myocardium of V1. The difference in integrated enhancement between V2 and V1 (dM_C_/dt) was then determined through image subtraction. After which, normalization by the average incoming aortic blood pool contrast concentration (C_IN_) and left ventricular myocardium tissue mass (M_T_) was performed to yield the average MS perfusion (P_AVG_MS_). Next, each voxel of the V1 myocardium was subtracted from each corresponding voxel of V2 (ΔHU) then normalized by the difference in average enhancement between the V2 and V1 myocardium (ΔHU_AVG_), yielding a perfusion ratio map. The average MS perfusion (P_AVG_MS_) was then multiplied by the perfusion ratio map (ΔHU/ ΔHU_AVG_) to yield voxel-by-voxel MS perfusion measurements (P_MI_).

Finally, virtual tissue plugs from the proximal LAD, distal LAD, and LCx perfusion territories were segmented, where these plugs were spatially matched to the physical tissue plugs using epicardial coronary landmarks. The per-voxel MI and MS perfusion values within each plug were then averaged and compared to corresponding microsphere perfusion measurements. The image processing steps are outlined in Fig. 1c,d.

### Statistical analysis

As multiple measurements were made per animal, the intra-cluster correlation of measurement was first computed and found to be 0.12. Hence, there was minimal correlation between intra-animal measurements, i.e., all measurements were treated as independent. Overall, MI and MS measurements were compared to microsphere measurements with t-testing, regression, Bland–Altman, Pearson’s correlation coefficient (r), Lin’s concordance correlation coefficient (CCC), root-mean-square-error (RMSE: accuracy), and root-mean-square deviation (RMSD: precision). The diagnostic performance of the MI and MS techniques in identification of functionally significant stenoses, i.e., LAD microsphere perfusion less than 1.0 mL/min/g at hyperemia^20^, was also assessed via sensitivity, specificity, positive and negative predictive values, accuracy, and area under the receiver operator characteristic curve (AUC). P-values less than 0.05 indicate significant differences. Statistical software was used (SPSS, Version 22, IBM Corporation).

## Results

### General

The average body mass of the six male Yorkshire swine was 37.3 ± 7.5 kg, where the average left ventricular mass of the swine was 52.1 ± 12.7 g. During image acquisition, the average heart rate of the swine was 73 ± 5 beats per minute, while their average mean arterial blood pressure was 73 ± 10 mmHg. Following image acquisition and image processing, the average time delay between the V1 and V2 volume scans was 4.7 ± 1.2 s, corresponding to an average change in myocardial enhancement over that time of 25.0 ± 13.5 HU.

For the LAD coronary artery, the average MI perfusion was 2.60 ± 1.94 mL/min/g, the average MS perfusion was 1.79 ± 1.34 mL/min/g, and the average microsphere perfusion was 2.62 ± 1.94 mL/min/g, where the result of corresponding t-testing was p = 0.72 and p = 0.00, respectively. For the LCx coronary artery, the average MI perfusion was 0.85 ± 0.40 mL/min/g, the average MS perfusion was 0.67 ± 0.56 mL/min/g, and the average microsphere perfusion was 0.87 ± 0.34 mL/min/g, where the result of corresponding t-testing was p = 0.75 and p = 0.05, respectively. Notably, LAD perfusion measurements were performed during intracoronary hyperemia (stress flow), while LCx perfusion measurements were not (rest flow).

### Accuracy and precision

**Figure 2:**
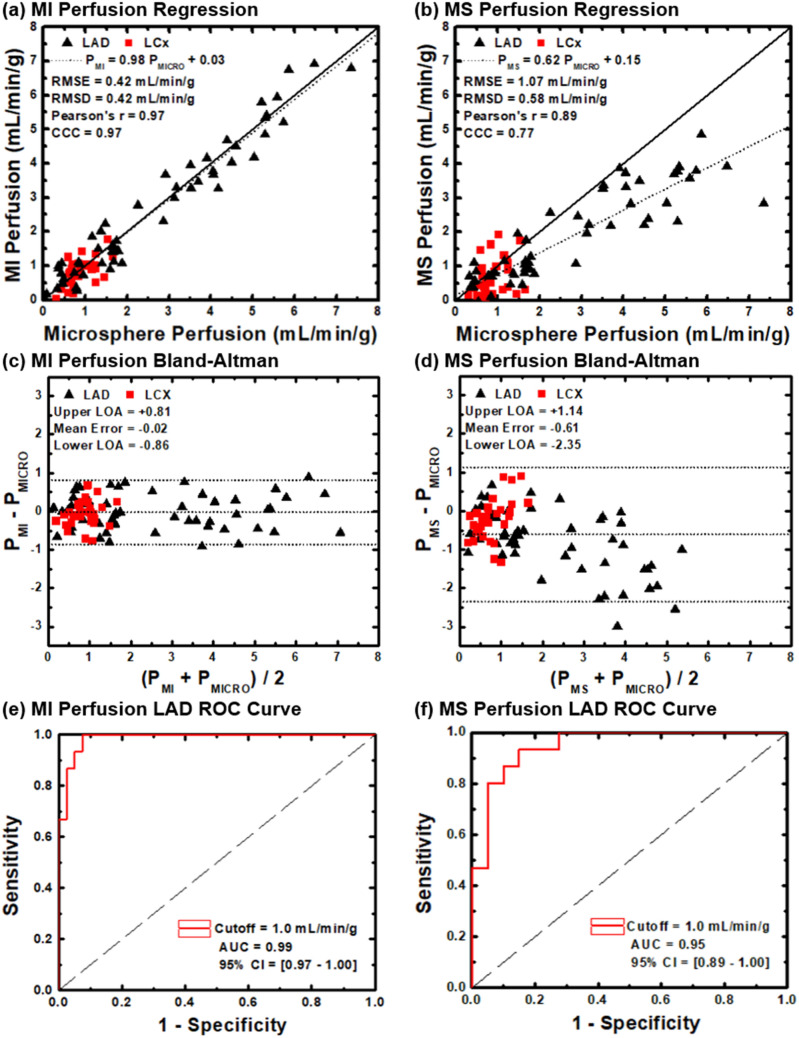
Accuracy and diagnostic performance. Regression analysis comparing (**a**) MI and (**b**) MS perfusion measurement to reference standard microsphere perfusion measurement. Bland–Altman analysis comparing (**c**) MI and (**d**) MS to reference standard microsphere perfusion measurement. AUC of the ROC for the (**e**) MI and (**f**) MS techniques in detection of functionally significant LAD stenosis, defined as stress perfusion less than 1.0 mL/min/g.

The regression equation relating MI perfusion (P_MI_) to microsphere perfusion (P_MICRO_) was P_MI_ = 0.98 P_MICRO_ + 0.03, where the Pearson’s and Lin’s correlations were r = 0.97 and ρ = 0.97, respectively, while the RMSE and RMSD were 0.42 mL/min/g and 0.42 mL/min/g, respectively. Alternately, the regression equation relating MS perfusion (P_MS_) to microsphere perfusion (P_MICRO_) was P_MS_ = 0.62 P_MICRO_ + 0.15, where the Pearson’s and Lin’s correlations were r = 0.89 and ρ = 0.77, respectively, while the RMSE and RMSD were 1.07 mL/min/g and 0.58 mL/min/g, respectively. All regression analyses are listed in Table 1 and displayed in Fig. 2, with corresponding Bland–Altman analyses also shown.

**Table 1:** Motion-immune (MI) and motion-susceptible (MS) CT perfusion as compared to reference standard microsphere perfusion.

Technique	Slope	Intercept	Pearson's r	Lin’s CCC	RMSE (mL/min/g)	RMSD (mL/min/g)
**MI perfusion (N)**						
LAD (55)	0.97** [0.91, 1.04]	0.05 [− 0.17, 0.26]	0.97** [0.95, 0.98]	0.97** [0.95, 0.98]	0.46	0.46
LCx (30)	0.66 [0.28, 1.04]	0.28 [− 0.08, 0.63]	0.56 [0.24, 0.76]	0.55 [0.23, 0.76]	0.35	0.33
LAD + LCx (85)	0.98** [0.92, 1.03]	0.03 [− 0.11, 0.17]	0.97** [0.96, 0.98]	0.97** [0.96, 0.98]	0.42	0.42
**MS Perfusion (N)**						
LAD (55)	0.62 [0.53, 0.71]	0.17 [− 0.11, 0.46]	0.89 [0.82, 0.93]	0.74 [0.59, 0.84]	1.27	0.61
LCx (30)	0.56 [− 0.04, 1.16]	0.18 [− 0.38, 0.74]	0.34 [− 0.02, 0.62]	0.28 [− 0.09, 0.58]	0.57	0.52
LAD + LCx (85)	0.62 [0.55, 0.69]	0.15 [− 0.04, 0.34]	0.89 [0.83, 0.92]	0.77 [0.67, 0.85]	1.07	0.58

Brackets indicate 95% confidence intervals.

*MI* motion-immune CT perfusion, *MS* motion-susceptible CT perfusion, *LAD* left anterior descending coronary artery, *LCx* left circumflex coronary artery, *N* number of perfusion measurements, *Lin’s CCC* Lin’s concordance correlation coefficient, *RMSE* root-mean-square error, *RMSD* root-mean-square deviation. **Indicates non-overlap of the 95% confidence intervals, i.e., significant differences between corresponding MI and MS parameters.

### Diagnostic performance

**Figure 3:**
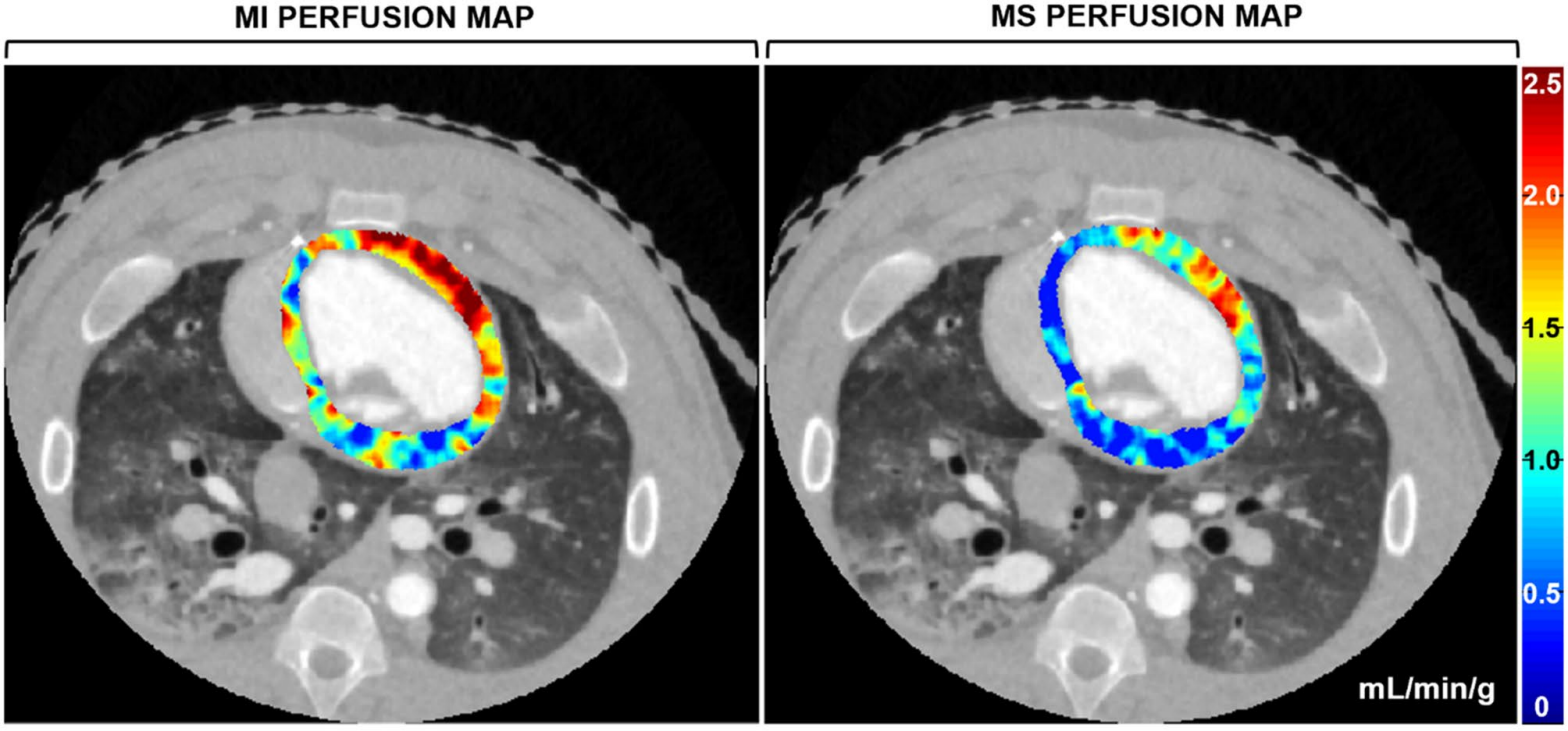
Motion-immune (MI) versus motion-susceptible (MS) perfusion mapping. For a single acquisition in the presence of motion without registration, MI perfusion mapping (left panel) produced accurate hyperemic perfusion measurements in the LAD territory, i.e., the anterior left ventricular wall, while corresponding MS perfusion mapping (right panel) underestimated perfusion measurements in the LAD territory, as well as globally. Images are displayed as axial views. The color bar indicates quantitative perfusion in milliliters per minute per gram of myocardium.

The sensitivity, specificity, and accuracy of the MI technique were 80%, 98%, and 93%, respectively, with an AUC of the ROC of 0.99. Whereas the sensitivity, specificity, and accuracy of the MS technique were 93%, 80%, and 84%, respectively, with an AUC of the ROC of 0.95. All diagnostic performance analyses are shown in Table 2 with each ROC displayed in Fig. 2. Example MI and MS perfusion maps are also shown in Fig. 3. Finally, the CT dose index was 10.8 mGy while the size-specific dose estimate was 17.8 mGy.

**Table 2:** Motion-immune (MI) and motion-susceptible (MS) CT perfusion-based detection of physiologically significant LAD stenosis.

Technique	SN (%)	SP (%)	PPV (%)	NPV (%)	ACCURACY (%)	ROC AUC
MI perfusion	80 (12/15) [52, 96]	98 (39/40) [87, 100]	92 (12/13) [63, 99]	93 (39/42) [83, 97]	93 (51/55) [82, 98]	0.99 [0.97, 1.00]
MS perfusion	93 (14/15) [68, 100]	80 (32/40) [64, 91]	64 (14/22) [48, 77]	97 (32/33) [83, 100]	84 (46/55) [71, 92]	0.95 [0.89, 1.00]

Parentheses indicate the fractional representation of measurements; Brackets indicate 95% confidence intervals. *MI* motion-immune CT perfusion, *MS* motion-susceptible CT perfusion, *LAD* left anterior descending coronary artery, *SN* sensitivity, *SP* specificity, *PPV* positive predictive value, *NPV* negative predictive value, *ROC AUC* area under the curve of the receiver operator characteristic.

## Discussion

### Indication of results

MI perfusion measurement agreed well with microsphere perfusion measurement, with near unity regression slope, excellent Pearson’s and Lin’s correlation, and minimal RMSE and RMSD. MI perfusion measurement also identified functionally significant stenoses with high accuracy, while only requiring two volume scans per measurement. Hence, accurate myocardial ischemia assessment, in the presence of motion without image registration, is feasible at a low dose (less than 3 mSv^15–18^).

### Comparison to previous reports

Notably, the MS perfusion technique was previously validated versus microsphere perfusion, using fully co-registered, motion-corrected data. As a result of registration, the RMSE, RMSD, and diagnostic performance were all improved as compared to the present MS technique without registration. Hence, image registration does improve the accuracy of dynamic CT perfusion measurement in the presence of motion between image acquisitions. However, the MI perfusion technique, in the absence of registration, demonstrated a lower RMSE and RMSD with better diagnostic performance than the MS perfusion technique both *with*^15^ and *without* registration; highlighting the inherent limitations of image registration. In particular, registration cannot accurately reproduce the true quantitative CT-number or position of a target following deformation, whereby the degree of inaccuracy increases as the magnitude of motion and required deformation increases^21,22^. As dynamic CT perfusion techniques rely on the change in myocardial CT number over many cardiac cycles, any process that modifies the true CT number, i.e., registration, motion, partial volume averaging, beam hardening, etc., will reduce the accuracy of perfusion measurement (via underestimation) leading to higher false positive rates, reduced specificity, and increased positive predictive value^14^. Of course, static CT perfusion techniques are less impacted, only displaying gantry rotational-motion induced blurring, but dynamic techniques perform superiorly in detection of intermediate severity CAD, especially under stress conditions^23,24^. Yet, even dynamic techniques, such as the maximum slope model, underestimate perfusion, since they rely on small tissue volumes-of-interest (VOI) for measurement, where these VOIs are subject to contrast mass entry and exit over the measurement time, especially at hyperemia. Whereas the MI technique defines the entire myocardium as a single large VOI, where measurements are made prior to hyperemic transit; thus, solving the problem of perfusion underestimation. Hence, the MI technique represents a “best-of-both-worlds” solution as it provides the diagnostic advantages of dynamic CT perfusion, while mathematically eliminating the negative impacts of motion and registration.

## Limitations

While referred to as “motion-immune,” it is important to note that the MI perfusion technique does not eliminate gantry motion blurring, which is inherent to cardiac CT image acquisition. Nevertheless, for motion between consecutive acquisitions caused by beat-to-beat variation (as in this study) or by imperfect breath-holding (to be studied in future work), the mathematics of the MI technique effectively eliminate the impact of such motion; thus, bypassing the need for and errors inherent to image registration. Additionally, the MI perfusion theory assumes that the unenhanced myocardial tissue density is relatively uniform within V1, i.e., that the average myocardial enhancement approximates the per-voxel enhancement with minimal variance. While this assumption holds true for patients without a prior history of myocardial infarction, chronically infarcted myocardium is known to be hypoattenuating secondary to reduced capillary density with increased adiposity^25^, where spotty myocardial calcifications may also be present (rarely)^26^. Hence, in the case of scar, both rest and stress perfusion may be underestimated, but the relation between coronary flow reserve and stress perfusion, i.e., coronary flow capacity, can be used to accurately discriminate scar from ischemia^27^.

Dynamic scanning was intentionally performed over many cardiac cycles to retrospectively develop and validate the two-volume MI technique. Hence, prospective validation remains necessary, and will require specialized acquisition timing of V1 and V2 at the base and peak of the aortic enhancement. Fortunately, a diluted test bolus protocol can be used, but requires extra contrast and radiation dose^28^. Alternatively, dynamic bolus tracking combined with a peak timing relation enables acquisition of V2 at approximately the aortic peak (within ± 2 cardiac cycles) while maintaining perfusion measurement accuracy^29^. In both cases, future clinical implementation will also require incorporation of breath-holding, to be initiated directly after contrast injection but before acquisition of V1, with exhalation after acquisition of V2. Meaning, V1 and V2 should be acquired during the same breath hold. Fortunately, such breath-holding is easily prompted on most clinical CT scanners and should not pose an issue to translation of the MI technique.

Additionally, prior work assessed perfusion in the LAD and LCx territories of the left ventricle along with the RCA territory of the right ventricle^15^, while this study only assessed perfusion in the LAD and LCx territories within the left ventricle. Specifically, image registration of V1 and V2 enables maximum intensity projection (MIP) image generation, where the resulting biventricular opacification can be used for segmentation of the main wall of the right ventricular for RCA perfusion measurements^15^. Without registration, however, only left ventricular segmentation can be performed. That said, if a triphasic injection protocol were to be employed, left and right ventricular opacification can be achieved within the V2 volume scan alone^30^, enabling biventricular perfusion measurement. Moreover, regarding the perfusion territories assessed, spatially matched virtual tissue plugs were used primarily for perfusion validation. In practice, however, minimum-cost-path perfusion territory or sub-territory assignment will be used for automatic delineation of the coronary perfusion territories, as previously validated^31^.

Finally, this study did not evaluate the impact of different registration algorithms on perfusion measurement accuracy. However, our prior CT perfusion results that employed image registration^15^ were discussed above. Additionally, as the raw data were previously acquired^15^, the limitations attributed to study design—central contrast injection, intracoronary adenosine, coronary balloon stenosis, small effective chest diameter, and acquisition timing—were all already addressed in detail and did not significantly impact the results of this work^15–18,29^.

## Conclusion

The motion-immune FPA perfusion technique provides a solution to dynamic CT perfusion measurement in the presence of motion without image registration. By eliminating image registration and using only two volume scans for dynamic CT perfusion measurement, the motion-immune perfusion technique can improve the quantitative accuracy of CT-based CAD risk assessment while also reducing the radiation dose.

## Data Availability

Available upon request to the Corresponding Author, Sabee Molloi.

## Abbreviations

CAD: Coronary artery disease
CCC: Lin’s concordance correlation coefficient
${CTDI}_{vol}^{32}$: 32 Cm diameter volumetric CT dose index
FFR: Fractional flow reserve
FPA: First-pass analysis
HU: Hounsfield unit
MI: Motion-immune FPA perfusion technique
MS: Motion-susceptible FPA perfusion technique
SSDE: Size-specific dose estimate
V1: Volume scan 1
V2: Volume scan 2
